# The Application of Piecewise Regularization Reconstruction to the Calibration of Strain Beams

**DOI:** 10.3390/s24092744

**Published:** 2024-04-25

**Authors:** Jingjing Liu, Wensong Jiang, Zai Luo, Penghao Zhang, Li Yang, Yinbao Cheng, Dian Bian, Yaru Li

**Affiliations:** 1 College of Metrology Measurement and Instrument, China Jiliang University, Hangzhou 310018, China; s21020804031@cjlu.edu.cn (J.L.); luozai@cjlu.edu.cn (Z.L.); cyb@cjlu.edu.cn (Y.C.); biandian155@cjlu.edu.cn (D.B.); liyaru@cjlu.edu.cn (Y.L.); 2 Changcheng Institute of Metrology & Measurement, Aviation Industry Corporation, Beijing 100095, China; zhangpenghao@buaa.edu.cn; 3 College of Information Engineering, China Jiliang University, Hangzhou 310018, China; 13a0303131@cjlu.edu.cn

**Keywords:** calibration, dynamic load reconstruction, ill-posed problem, piecewise Tikhonov regularization

## Abstract

Standard beams are mainly used for the calibration of strain sensors using their load reconstruction models. However, as an ill-posed inverse problem, the solution to these models often fails to converge, especially when dealing with dynamic loads of different frequencies. To overcome this problem, a piecewise Tikhonov regularization method (PTR) is proposed to reconstruct dynamic loads. The transfer function matrix is built both using the denoised excitations and the corresponding responses. After singular value decomposition (SVD), the singular values are divided into submatrices of different sizes by utilizing a piecewise function. The regularization parameters are solved by optimizing the piecewise submatrices. The experimental result shows that the MREs of the PTR method are 6.20% at 70 Hz and 5.86% at 80 Hz. The traditional Tikhonov regularization method based on GCV exhibits MREs of 28.44% and 29.61% at frequencies of 70 Hz and 80 Hz, respectively, whereas the L-curve-based approach demonstrates MREs of 29.98% and 18.42% at the same frequencies. Furthermore, the PREs of the PTR method are 3.54% at 70 Hz and 3.73% at 80 Hz. The traditional Tikhonov regularization method based on GCV exhibits PREs of 27.01% and 26.88% at frequencies of 70 Hz and 80 Hz, respectively, whereas the L-curve-based approach demonstrates PREs of 29.50% and 15.56% at the same frequencies. All in all, the method proposed in this paper can be extensively applied to load reconstruction across different frequencies.

## 1. Introduction

Standard beams serve as important testing tools in engineering practice, widely used in fields such as mechanical performance testing and sensor calibration [1,2,3]. Their main function is to act as a load reconstruction model in the process of sensor calibration. Load reconstruction commonly employs the direct inversion method. In this scenario, there are equal numbers of equations and unknowns. However, the singular values of the transfer function matrix are very close to zero, rendering the problem ill posed. Solving ill-posed inverse problems is often challenging. This challenge is particularly evident at different signal frequencies, as frequency variations may significantly affect the reconstruction results. To address the fact that the load reconstruction problem is ill posed, appropriate numerical analytical methods are adopted [4]. This includes methods such as probabilistic statistical methods and regularization methods [5,6].

In terms of the first type of method, the direct inverse method can reconstruct a dynamic load based on the relationship between the dynamic load and the measured response of the structure. For example, Liu et al. [7] reconstructed the load directly by solving a small-scale regular linear system in m dimensions. Zhao et al. [8] obtained the energy directly to determine the structural damage using wavelet packets. Although this kind of method is simple and easy to access, the fact that the direct inverse relationship is ill posed can decrease the accuracy of the reconstruction with an unknown uncertainty.

To avoid this problem, probabilistic/statistical methods are investigated. This utilizes known prior probability information to estimate unknown quantities, thereby alleviating the fact that the problem is ill posed. For example, Bayesian methods have been investigated by many scholars for reconstructing an impact load [9,10,11]. Prawin and Rama Mohan Rao [12] proposed an online load reconstruction algorithm based on dynamic principal component analysis with overlapping moving windows. Jiang et al. [13] achieved reconstruction of an impact load by redefining the transfer function of the reconstruction model using a Fractional-Order Accumulative Regularization Filter. Pallekonda et al. [14] used an Artificial Neuro-Fuzzy Inference System for load reconstruction. Another common type of adaptive estimation is the Kalman filter, which is widely used in load reconstruction techniques [15,16,17,18]. However, the uncertainties existing in the actual situation are difficult to solve in advance, and this kind of method cannot be applied to all load reconstruction processes with a fixed model.

To solve these difficulties, regularization methods are proposed to improve the fact that the load reconstruction problem is ill posed by optimizing singular values in the transfer function matrix [19]. For example, Lu et al. [20] proposed a novel dynamic load identification method based on the Least Squares Time Element Method (LSTEM), a wavelet scaling function, and a regularization method. Miao et al. [21] proposed a finite element modification model combined with the Tikhonov regularization method for the reconstruction of a periodic load. Tang et al. [22] proposed a Tikhonov regularized total least squares method and verified the load reconstruction on an aluminum plate. Sun et al. [23] combined matrix equations and regularization methods to derive unbalanced forces based on vibration responses. He et al. [24] proposed detailed load bound identification methods to identify an uncertain structural load in the frequency domain. Miao et al. [25] conducted a comparative analysis of different regularization methods in terms of their accuracy, noise immunity, and robustness.

Equally, a series of improved regularization methods have been investigated. For example, Wang et al. [26] proposed the conjugate gradient method, Aucejo and De Smet [27] proposed the iterative multiplication method, and Zheng et al. [28] and Chang et al. [29] proposed the Landweber iterative method. Chen et al. [30] and Yang et al. [31] applied optimization algorithms to the identification of moving dynamic loads. Adding sparsity is a good way to reduce the complexity of the problem, and sparse regularization [32] and nonconvex regularization [33,34,35,36] have received increasing attention. To reduce the number of unknowns and improve the speed and accuracy of the solution, Tran and Inoue [37] used wavelet bases for impact load reconstruction. The regularization method is a common method used to realize dynamic load reconstruction. Although this method can optimize the ill-posed solutions caused by small singular values, it can also generate model errors by suppressing large singular values. This situation will result in larger errors under signals of different frequencies.

Aiming at improved load reconstruction performance under different frequency signals, a piecewise Tikhonov regularization method is proposed, combined with probabilistic statistical methods. This method not only addresses the issues caused by small singular values but also strives to retain important large singular values, thus balancing the accuracy and stability of the approximate solution. The core of this approach lies in the rational partitioning of singular values and determining the appropriate regularization parameters to tackle load reconstruction problems under different frequencies.

The remainder of this paper is organized as follows. In Section 2, a PTR model for an ill-posed problem is developed. In Section 3, numerical simulations are conducted to assess how ill posed the model is. In Section 4, excitations on a cantilever beam are reconstructed using the PTR method. The reconstruction accuracy of the PTR method is compared with the traditional Tikhonov regularization method. Finally, several conclusions and research expectations about our work are given in Section 5.

## 2. Overview of the PTR Model

**Figure 1:**
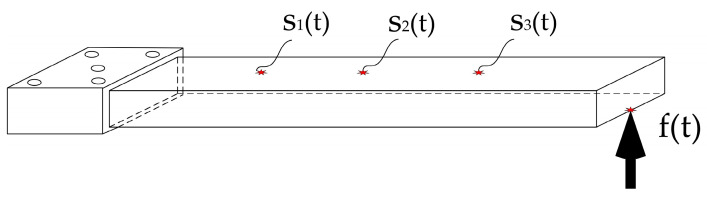
Structure of the aluminum alloy cantilever beam. The responses are denoted by S_1_(t), S_2_(t), and S_3_(t), and the excitation load is denoted by f(t).

To numerically represent the reconstruction principles for a dynamic load, an aluminum alloy cantilever beam is designed, as Figure 1 shows. Considering that the transfer function matrix is approximately singular, a segmented optimization regularization method with singular values is proposed to improve the fact that the problem is ill posed. The proposed PTR method includes denoising the initial signal using Fourier transformation, modeling the transfer function matrix of the test body, performing SVD on the transfer function matrix, segmenting the regularization of singular values, and dynamically reconstructing dynamic loads based on the measured responses.

To minimize the noise from the signal acquisition system, denoising operations are applied in the time domain to dynamic loads f(t) and their responses s(t), that is:

(1) $$\{\begin{array}{c} f(t)=\frac{a_{0}}{2}+\sum_{n=1}^{\infty }(a_{n}cos\frac{n\pi t}{L}+b_{n}sin\frac{n\pi t}{L})\\ s(t)=\frac{a_{0}}{2}+\sum_{n=1}^{\infty }(a_{n}cos\frac{n\pi t}{L}+b_{n}sin\frac{n\pi t}{L}) \end{array}$$

where f(t) is an excitation, s(t) is a response, L is a constant, a_0_ is the average value of f(t) or s(t) over a cycle, $a_{n}$ and $b_{n}$ are the amplitudes of the n-th harmonic, and n is the number of harmonics.

Assuming that $t_{i}=\Delta t\times i$, Δt is the discrete sampling interval in time history t; in $t_{i}$-th time, the discrete response signal is $s_{i}=s\left(t_{i}\right)$; and the discrete load signal is $f_{j}=f\left(t_{j}\right)$. In the time domain, the matrix relationship between the discrete input and output can be expressed as:

(2) $$\left(\begin{array}{l} s_{1}\\ s_{2}\\ \vdots \\ s_{m} \end{array}\right)=\left(\begin{array}{cccc} g_{1} & & & \\ g_{2} & g_{1} & & \\ \vdots & \vdots & \ddots & \\ g_{m} & g_{m-1} & \cdots & g_{1} \end{array}\right)\left(\begin{array}{l} f_{0}\\ f_{1}\\ \vdots \\ f_{m-1} \end{array}\right)$$

where m is the number of samples in the time domain for a single excitation and a single response system. Equation (2) can be simplified as:

(3) S=G×F

where **G** is a transfer function matrix in the time domain. It can be seen from Equation (3) that **G** is determined by the structural characteristics of the aluminum alloy cantilever beam.

The SVD is applied to decompose the transfer function matrix **G** into its singular value and vector components, that is:

(4) $$G=U(\mathrm{diag}(\sigma_{i}))V^{T}$$

where $U=\left(u_{1},u_{2},\cdots ,u_{m}\right)$ and $V=\left(v_{1},v_{2},\ldots ,v_{m}\right)$. $V^{T}$ is the transpose of **V**. $u_{i}$ and $v_{i}$ are the left and right singular vectors, respectively. $\sigma_{i}$ is a singular value, and $\mathrm{diag}(\sigma_{i})$ is a singular matrix.

In practical measurement situations, the actual responses **S** are generally interfered with by noise $\delta =[\delta_{i}|i=1,2,\cdots ,m]$, which will lead to an inequality between the actual responses **S** and the measured responses $S^{\delta }$, i.e., $S^{\delta }=S+\delta$.

The load can be reconstructed by coupling Equations (3) and (4) after simple algebraic manipulations, that is:

(5) $$F^{\delta }=G^{-1}\cdot S^{\delta }=V\mathrm{diag}(\sigma_{i}^{-1})U^{T}\cdot S^{\delta }=F_{\mathrm{tr}}+\sum_{i=1}^{m}\sigma_{i}^{-1}(u_{i}^{T}\delta )v_{i}$$

where **G**^−1^ is the inverse matrix of **G**, and **F**_tr_ is the actual load. Equation (5) implies that a small perturbation can amplify the error of the reconstructed load with the help of the singular values in **G**. This will lead to the ill-posed inverse problem that $F^{\delta }$ is quite different from **F**_tr_. To solve this ill-posed inverse problem, the singular matrix should be corrected appropriately.

To overcome the fact that the transfer function matrix **G** is ill posed, the singular matrix is corrected by utilizing Tikhonov filter factors. The load $F^{\alpha ,\delta }$ can be reconstructed using the traditional Tikhonov regularization method as:

(6) $$F^{\alpha ,\delta }=V\mathrm{diag}\left(f(\alpha ,\sigma_{i})\cdot \sigma_{i}^{-1}\right)U^{T}\cdot S^{\delta }=\sum_{i}\frac{f(\alpha ,\sigma_{i})}{\sigma_{i}}(u_{i}^{T}S^{\delta })v_{i}$$

where α is a regularization parameter, and $f(\alpha ,\sigma_{i})$ is the extended Tikhonov filter factor, which is:

(7) $$f(\alpha ,\sigma_{i})=\sigma_{i}\times {\left(\sigma_{i}+\alpha \right)}^{-1}$$

It can be observed from Equation (5) that the instability of the problem is mainly caused by the small singular values of the transfer function matrix. As these singular values approach zero, their inverse tends toward infinity, leading to a significant amplification of the noise in the response. Therefore, starting from the global processing approach of the traditional methods, a new segmented regularization method is proposed. The singular values are divided into K parts, and different amplitude singular values are processed differently to reduce the amplification of the noise. The PTR method is expressed as:

(8) $$F^{\alpha ,\delta }=\sum_{k=1}^{K}\sum_{i=P_{k}}^{Q_{k}}\frac{f_{k}(\alpha_{k},\sigma_{i})}{\sigma_{i}}(u_{i}^{T}S^{\delta })v_{i}$$

Singular values are divided into K pieces using Equation (8) in descending order. P_k_ and Q_k_ are the minimum limit position and maximum limit position in the kth piece, respectively, and:

(9) $$\sum_{k=1}^{K}\left(Q_{k}-P_{k}+1\right)=m$$

where m = rank(**G**). The maximum number of divisions K_1_ is determined based on the order of magnitude of the singular values, and the decision strategy is designed as:

(10) $$K_{1}=\left\lfloor 1+lg\left(\frac{\sigma_{1}}{\sigma_{m}}\right)\right\rfloor$$

Set $\gamma_{1}\cdots \gamma_{k}$ if:

(11) $$\frac{\sigma_{1}-\sigma_{i}}{\sigma_{1}-\sigma_{m}}\geq \gamma_{k}$$

Then, set:

(12) $$\begin{array}{cc} P_{k+1}=i, & Q_{k}=i \end{array}-1$$

where ⌊⋅⌋ is a lower bound operation, $\gamma_{k}$ is the segmentation parameter at the k-th and (k + 1)-th segments, and $\gamma_{k}=0.1,0.2\cdots 1$. In particular, P_1_ corresponds to the position of $\sigma_{1}$, and Q_K_ corresponds to the position of $\sigma_{m}$.

The traditional methods use techniques such as generalized cross-validation and L-curves to solve the regularization parameters. However, the regularization parameters $\alpha_{k}$ of the method proposed in this paper can be solved using an unconstrained simplex search algorithm. The average relative error of $F^{\alpha ,\delta }$ and $F^{\delta }$ is the optimization objective. The objective function for the regularization parameter $\alpha_{k}$ is:

(13) $$\underset{\alpha_{k}}{\min}(\mathrm{mean}(\frac{\mathrm{abs}(F^{\alpha ,\delta }-F^{\delta })}{F^{\delta }}))=\underset{\alpha_{k}}{\min}(\mathrm{mean}(\mathrm{abs}(\sum_{k=1}^{K}\sum_{i=P_{k}}^{Q_{k}}\frac{f_{k}(\alpha_{k},\sigma_{i})}{\sigma_{i}}(u_{i}^{T}S^{\delta })v_{i}-F^{\delta })))$$

where $K\leq K_{1}$, abs(⋅) is an absolute value operation, mean(⋅) is an averaging operation, and mean(⋅) is a minimization operation.

By substituting Equations (7) and (13) into Equation (8), the load on the source point can be reconstructed as:

(14) $$F^{\alpha ,\delta }=\sum_{k=1}^{K}\sum_{i=P_{k}}^{Q_{k}}{\left(\sigma_{i}+\alpha_{k}\right)}^{-1}(u_{i}^{T}S^{\delta })v_{i}$$

**Figure 2:**
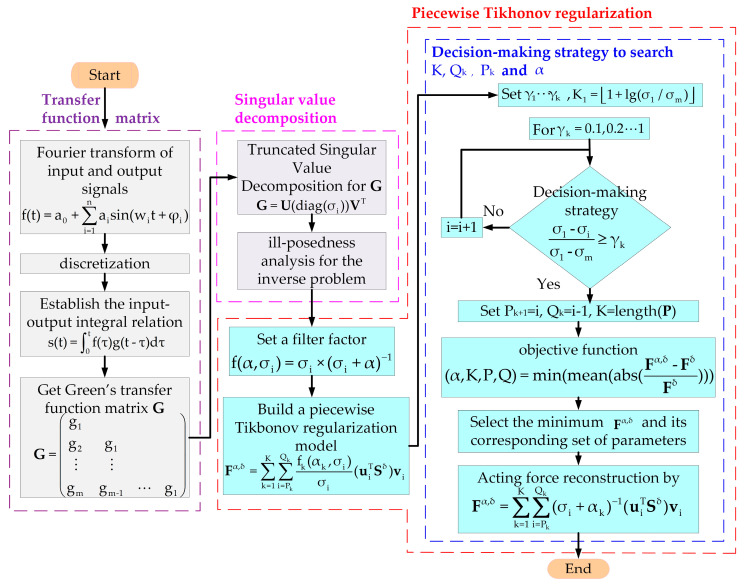
Flow chart of the PTR method.

All in all, after Fourier series fitting and PTR, the exciting load can be reconstructed using Equation (14), as Figure 2 shows.

## 3. Numerical Simulations

**Figure 3:**
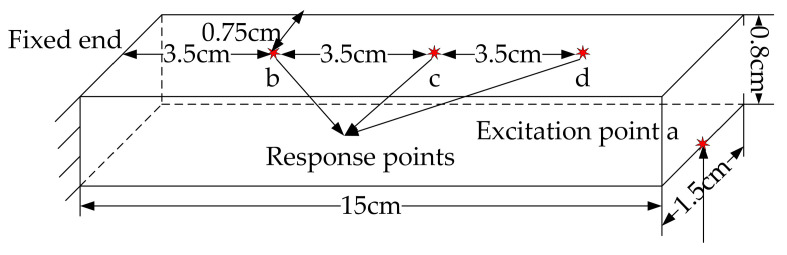
Test schematic.

The purpose of numerical simulations is to solve the initial transfer function matrix and evaluate how ill posed the tested beam structure is. The cantilever beam structure is designed. In detail, the beam length is 1.50 × 10^1^ cm, the width is 1.50 cm, the thickness is 8.00 × 10^−1^ cm, the density of the aluminum alloy is 2.77 × 10^3^ kg∙m^−3^, its modulus of elasticity is 7.1 × 10^1^ GPa, and its Poisson’s ratio is 0.33. The primary constraint of this structure is the unilateral fixed support; specifically, one end of the cantilever beam is affixed while the other remains free. The boundary conditions at the fixed end of the cantilever beam are expressed as *u*(0) = 0, *v*(0) = 0, *θ*(0) = 0, where u and v denote the linear displacements along the x and y axes, respectively, and θ represents the rotational angle around the z-axis. The initial transfer function matrix can be solved using finite element simulation in Ansys Workbench. Considering the current experimental setup and the inherent frequency of the system, a specific series of sinusoidal loads ranging from 40 Hz to 90 Hz are selected for dynamic stimulation. The load is applied at point a on the cantilever beam. The responses are concurrently measured at points b, c, and d using FBG strain sensors. The geometric interrelationships among these points are explicitly outlined. A schematic diagram of the simulation test is shown in Figure 3.

**Figure 4:**
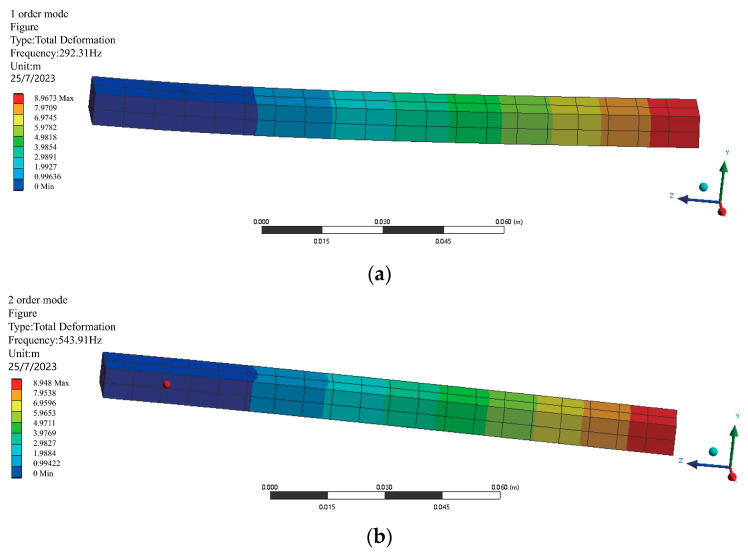
Mode shapes. (**a**) The translation mode in the y-direction; (**b**) the translation mode in the x-direction.

(1) The characteristics of the mode are simulated to analyze the resonance of the cantilever beam. The first- and second-order modal parameters of the cantilever beams are 292.31 Hz and 543.36 Hz. Resonance may occur when the frequency of the external excitations is close to the intrinsic frequency of the system. It can be seen that both the 1st order and the 2nd order of the modal are not equal to the selected frequencies at 40 Hz, 50 Hz, 60 Hz, 70 Hz, 80 Hz, and 90 Hz. This indicates that the selected frequencies are quite suitable for numerical simulations. The corresponding mode shapes are shown in Figure 4. (**a**) represents the cantilever beam translated in the y-direction, and (**b**) represents the cantilever beam translated in the x-direction.

A sinusoidal load with an amplitude of 100 N, a phase of 0 rad, and frequencies of 40 Hz, 50 Hz, 60 Hz, 70 Hz, 80 Hz, and 90 Hz are acted on point a, respectively. Modal superposition is used to obtain the responses at points b, c, and d. The measured responses are described as:

(15) $$S^{\delta }=S+10^{-0.05l^{\delta }}\times \mathrm{std}(S)\times \mathrm{randn}$$

where **S** is the response calculated using the simulation, std(S) is the standard deviation of the calculated response, $l^{\delta }$ is the signal-to-noise ratio (SNR), and randn is a white noise random number of the same length as S, which satisfies a normal distribution.

(2) To illustrate the impacts of noise on the ill-posed solution, a condition number is introduced. The condition number K of a matrix **G** is:

(16) $$K(G)=\left\|G^{-1}\right\|\times \left\|G\right\|$$

where **G** is a transfer function matrix, and **G**^−1^ is the inverse of **G**.

There are three kinds of condition numbers, which include the H1, H2, and H∞ condition numbers. A condition number is used to evaluate whether or not a function is ill posed. In detail, if the condition number of a matrix is quite big, then the function is ill posed; otherwise, it is a well-posed function. To assess how well posed this issue is, the condition numbers of transfer function matrices constructed from signals of different frequencies at various signal-to-noise ratios are compared, as Table 1 shows. If the condition number shows no significant variation across different signal-to-noise ratios, the problem is well posed; otherwise, it is ill posed.

The common matrix H2 is used here. It can be observed from Table 1 that the condition numbers exhibit a consistent pattern across all frequencies. The condition number is 1 when the SNR = ∞. However, the condition number becomes high when the SNRs are 26 dB, 20 dB, and 14 dB, respectively. This indicates that the model is ill posed due to noise.

**Figure 5:**
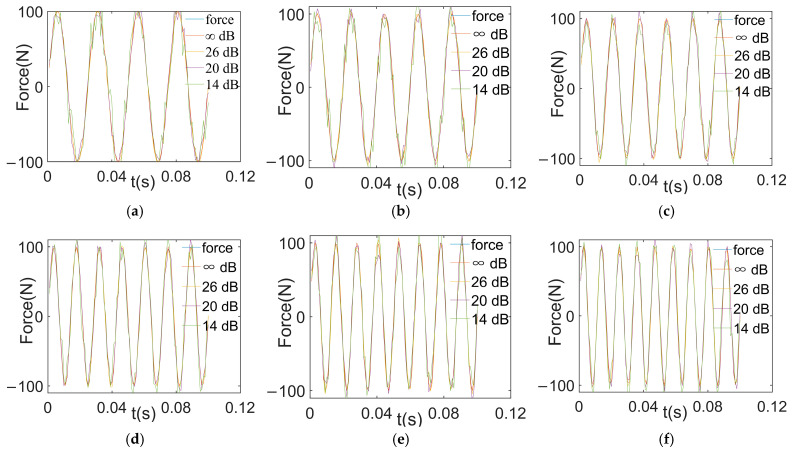
Reconstructed loads with different SNRs. (a) Reconstructed excitation at 40 Hz; (b) reconstructed excitation at 50 Hz; (c) reconstructed excitation at 60 Hz; (d) reconstructed excitation at 70 Hz; (e) reconstructed excitation at 80 Hz; (f) reconstructed excitation at 90 Hz.

(3) To illustrate the error amplification effects of noise on the ill-posed model, dynamic loads with different SNRs are reconstructed. The excitations are reconstructed from the responses with different SNRs, as Figure 5 shows.

There are four indexes introduced to evaluate the error of the load reconstruction, as follows:

(1) Relative error in time history (RE)

(17) $$e_{\mathrm{ss}}=\frac{\left|F^{\prime }-F\right|}{F}\times 100\%$$

(2) Mean relative error (MRE)

(18) $$\mathrm{MRE}=\mathrm{mean}(e_{\mathrm{ss}})$$

(3) Peak relative error (PRE)

(19) $$\mathrm{PRE}=\frac{\left|\max(F^{\prime })-\max(F)\right|}{\max(F)}\times 100\%$$

(4) Correlation (Cor)

(20) $$\mathrm{Cor}=\frac{\sum_{t=1}^{N}\left(F_{t}^{\prime }-\bar{F^{\prime }})(F_{t}-\bar{F}\right)}{\sqrt{\sum_{t=1}^{N}{\left(F_{t}^{\prime }-\bar{F^{\prime }}\right)}^{2}\sum_{t=1}^{N}{\left(F_{t}-\bar{F^{\prime }}\right)}^{2}}}$$

where $F^{\prime }$ is a reconstructed excitation; **F** is an initial excitation; and |⋅| is an absolute value operation. The value of Cor is between −1 and 1. Cor=−1 means that $F^{\prime }$ is perfectly negatively correlated with F; Cor=1 means that $F^{\prime }$ is perfectly positively correlated with F; and Cor=0 means that $F^{\prime }$ is not correlated with F.

The values of the MRE and PRE determine how ill posed the problem is, as Table 2 shows. It can be seen that the MRE and PRE are smaller when the reconstruction result is closer to the real load. Table 3 shows the Cor values. It can be seen that when the Cor is high, the MRE and PRE are small in theory. However, the MRE and PRE are amplified when they are affected by noise.

According to Table 2, the MRE and PRE show a growing trend as the SNR decreases. This shows that the SNR is negatively correlated with the reconstruction results. According to Table 3, as the SNR decreases, the Cor is maintained above 0.97. It indicates that the reconstruction results are all highly correlated. Overall, these indicate that noise has an amplifying effect on errors in an ill-posed model.

## 4. Experiment and Discussion

### 4.1. Experimental Setup

**Figure 6:**
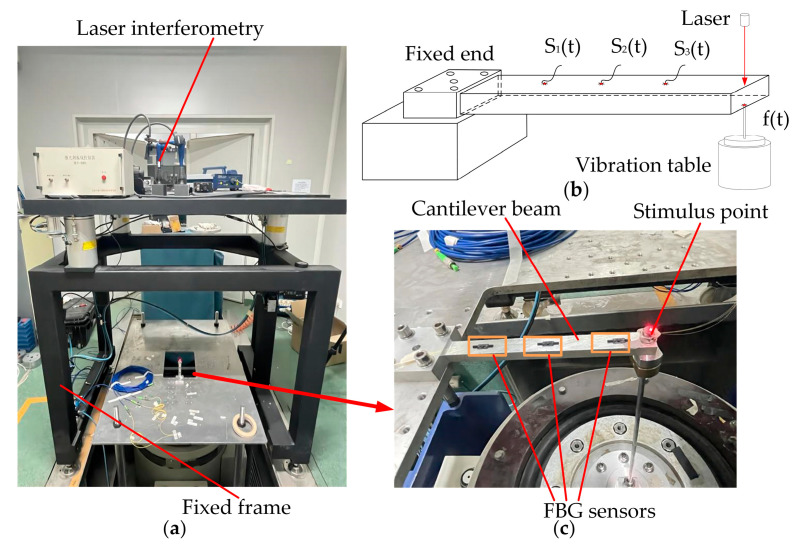
Load reconstruction model. (**a**) Experimental setup; (**b**) detailed diagram; (**c**) 3D structural schematic.

To illustrate the advantages of the application of the PTR method to dynamic load reconstruction, an experiment is carried out on a standard cantilever beam by utilizing a sinusoidal load. The experimental system consists of a cantilever beam, three FBG strain transducers, a shaker, a vibration isolation system, a laser interferometry system, and a data analysis system, as Figure 6 shows. The selected cantilever beam is an aluminum alloy with a length of 15.00 cm, a width of 1.50 cm, a thickness of 0.80 cm, a modulus of elasticity of E1 = 7.10 × 10^1^ Gpa, a density of P = 2.77 × 10^3^ kg∙m^−3^ and Poisson’s ratio of 0.33. One end of the beam is fixed onto a horizontal workbench. The selected laser interferometer is a PSV-400 with an accuracy of 1%. An FBG strain sensor is installed at each of points b, c, and d of the beam equidistantly.

There are four steps for our designed experiment, as follows,

1st. Signal processing. The activated shaker vibrates the free end of the cantilever beam. The excitations and the corresponding responses are collected and treated in Fourier series using Equation (1).

2nd. Modeling. The initial transfer function matrix is solved using Equation (2) based on the excitations and the corresponding responses at 70 Hz and 80 Hz.

3rd. Dynamic load reconstruction. To validate the effectiveness of the PTR method, dynamic loads at 40 Hz, 50 Hz, 60 Hz, and 90 Hz are reconstructed and evaluated. In the process of regularization, the segmentation bounds are determined using Equations (9)–(13), and the regularization parameters are solved using Equation (13). The singular matrix of the transfer function matrix **G** is corrected using Equation (7). According to the denoising responses and the modified singular matrix, the load can be reconstructed using Equation (14).

4th. Evaluation. Evaluation indexes such as the RE, MRE, PRE, and Cor are solved to comprehensively evaluate the reconstruction capability of the suggested PTR method.

The sinusoidal excitations are applied to the free end, and the resulting velocity signals are collected using the laser interferometer. Then, we can solve the velocity of the free end as:

(21) v(t)=U×a

where U is the voltage signal output by the laser interferometer, a is the sensitivity of the sampling signals, and a = 50.

### 4.2. Data Analysis and Error Evaluation

Considering the current experimental setup and the inherent frequency of the system, a specific series of sinusoidal loads ranging from 40 Hz to 90 Hz is still selected for dynamic stimulation.

**Figure 7:**
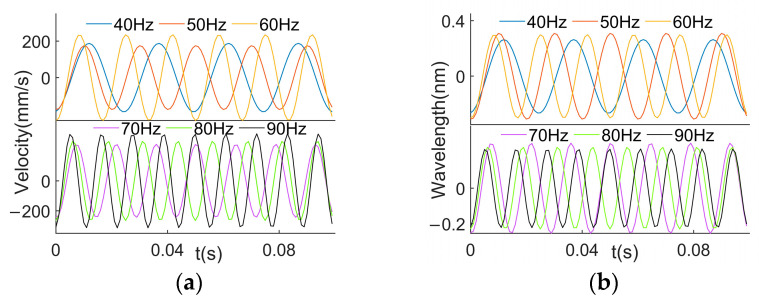
Signals. (**a**) Excitations; (**b**) responses.

The analysis of the transfer function matrix in the numerical simulation section indicates that the cantilever beam’s transfer function matrix amplifies the errors caused by noise. Fourier series fitting enables compression and denoising. Therefore, to minimize the impact of external noise on the reconstructed results, a Fourier series is employed for the signal fitting. The time constant of the strain sensor is estimated to be 1 ms according to the experiments, so the Fourier analysis time step is set to 1 ms in this paper. The results of the signal fitting are shown in Figure 7. It shows that the signal is smoother after denoising.

**Figure 8:**
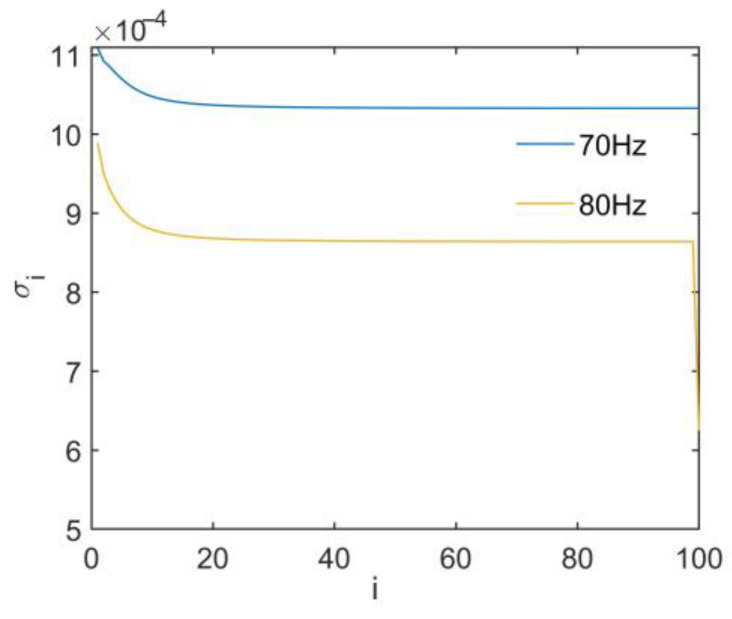
Singular value.

Compared with the traditional Tikhonov regularization method, the PTR method changes the optimization strategy for singular values. This avoids the over-regularization and under-regularization caused by the correction of the same regularization parameters. The initial transfer function matrices built at 70 Hz and 80 Hz are used for SVD, as Figure 8 shows. This shows that the PTR method should be used for optimization when singular values mutate.

There are many traditional load reconstruction methods. Here, to investigate the effectiveness of the PTR method, a comparison is made between the PTR method and the traditional Tikhonov regularization method based on generalized cross-validation (GCV) and the traditional Tikhonov regularization method based on an L-curve.

**Figure 9:**
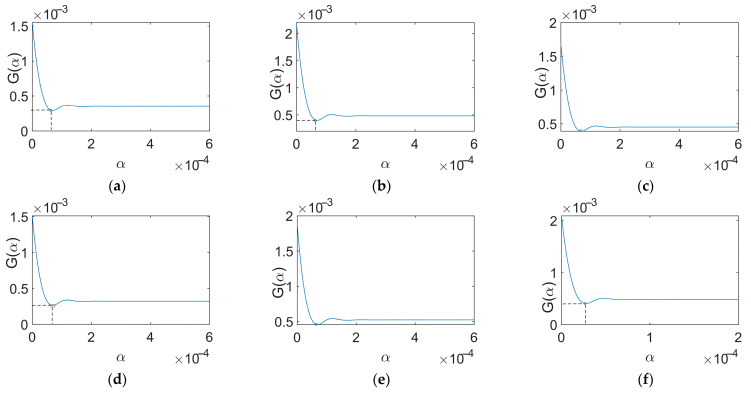
GCV curves (70 Hz). (**a**) 40 Hz; (**b**) 50 Hz; (**c**) 60 Hz; (**d**) 70 Hz; (**e**) 80 Hz; (**f**) 90 Hz.

**Figure 10:**
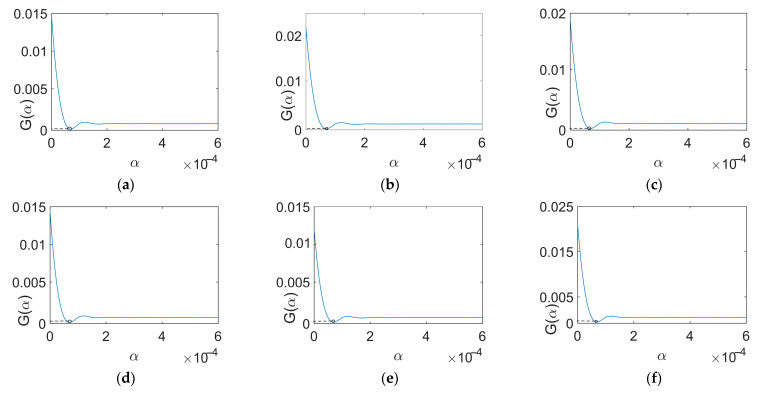
GCV curves (80 Hz). (**a**) 40 Hz; (**b**) 50 Hz; (**c**) 60 Hz; (**d**) 70 Hz; (**e**) 80 Hz; (**f**) 90 Hz.

The GCV curves derived from the signals ranging between 40 Hz and 90 Hz with an initial value of 70 Hz correspond to Figure 9. The GCV curves derived from the signals ranging between 40 Hz and 90 Hz with an initial value of 80 Hz correspond to Figure 10. The circles mark the points representing the optimal regularization parameter values.

**Figure 11:**
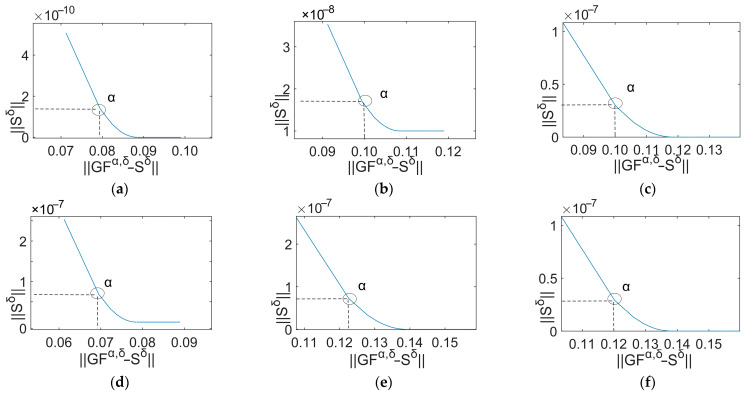
L-curves (70 Hz). (**a**) 40 Hz; (**b**) 50 Hz; (**c**) 60 Hz; (**d**) 70 Hz; (**e**) 80 Hz; (**f**) 90 Hz.

**Figure 12:**
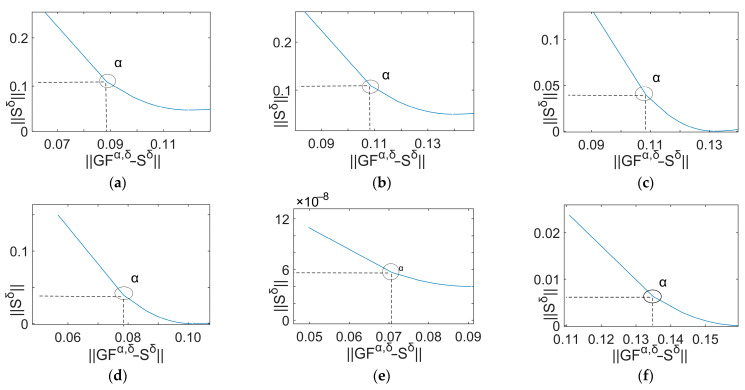
L-curves (80 Hz). (**a**) 40 Hz; (**b**) 50 Hz; (**c**) 60 Hz; (**d**) 70 Hz; (**e**) 80 Hz; (**f**) 90 Hz.

It can be observed that the optimal regularization parameter lies at the minimum point of the curve. The L-curves derived from the signals ranging between 40 Hz and 90 Hz with an initial value of 70 Hz correspond to Figure 11. The L-curves derived from the signals ranging between 40 Hz and 90 Hz with an initial value of 80 Hz correspond to Figure 12. The circles mark the points representing the optimal regularization parameter values.

It can be seen that the optimal regularization parameter lies at the turning point of the curves. To further study whether the number of segments is reasonable or not, the number of segments is increased based on the PTR method. PTR_2 increments the number of segments based on the PTR_1 method by 1. According to the objective function formulated in Equation (13), the segmentation and regularization parameters are computed. The segmentation parameters are shown in Table 4.

From Table 4, it can be seen that the PTR_1 and PTR_2 methods divide the singular values into two and three subsets, respectively. Combined with the distribution of singular values shown in Figure 8, it can be observed from Table 4 that the segment boundary points appear near the inflection points of the singular values.

**Figure 13:**
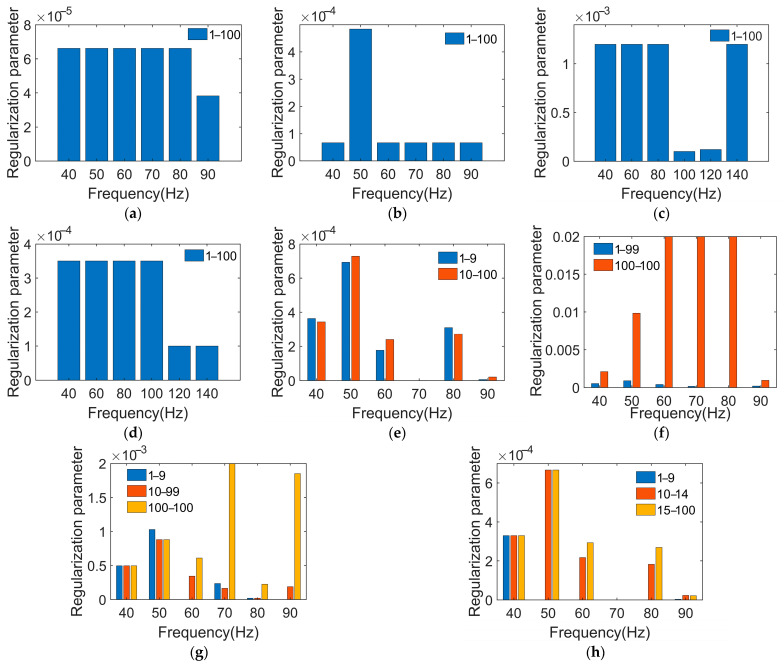
The regularization parameters of different transfer function matrices are optimized using different regularization methods. (**a**) Traditional Tikhonov regularization method based on GCV at 70 Hz; (**b**) traditional Tikhonov regularization method based on GCV at 80 Hz; (**c**) traditional Tikhonov regularization method based on L-curve at 70 Hz; (**d**) traditional Tikhonov regularization method based on L-curve at 80 Hz; (**e**) PTR_1 at 70 Hz; (**f**) PTR_1 at 80 Hz; (**g**) PTR_2 at 70 Hz; (**h**) PTR_2 at 80 Hz.

The solved regularization parameters are shown in Figure 13. The regularization parameters of the traditional Tikhonov regularization method can be determined using the GCV technique, as shown in Figure 13a,b. The regularization parameters derived from the L-curve are shown in Figure 13c,d. The regularization parameters obtained using the optimization algorithm are shown in Figure 13e,h. In mathematics, larger regularization parameters impose greater penalties on singular values, resulting in smoother and more stable solutions. Conversely, smaller regularization parameters reduce the penalties on singular values, potentially increasing the instability of and the risk of overfitting in the solution. Therefore, appropriate selection of the regularization parameters is crucial to both the accuracy and stability of the model.

The distribution of the regularization parameters shows that small singular values correspond to large regularization parameters. Comparing the PTR method with the traditional Tikhonov regularization method using GCV and L-curves, the model errors caused by singular values are corrected more efficiently. This leads to more accurate and stable reconstruction results. However, it can be seen from Figure 13g,h that the equality of the regularization parameters indicates that the segmentation is not effective.

**Figure 14:**
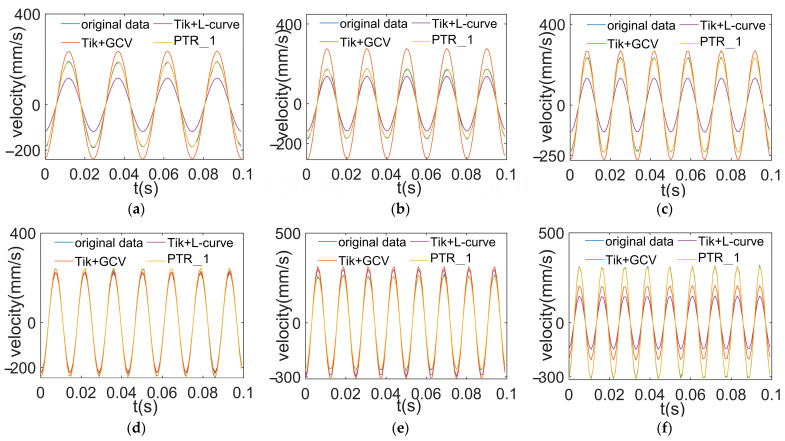
Reconstructed load at different frequencies using an initial value of 70 Hz. (**a**) Reconstructed excitation at 40 Hz; (**b**) reconstructed excitation at 50 Hz; (**c**) reconstructed excitation at 60 Hz; (**d**) reconstructed excitation at 70 Hz; (**e**) reconstructed excitation at 80 Hz; (**f**) reconstructed excitation at 90 Hz.

**Figure 15:**
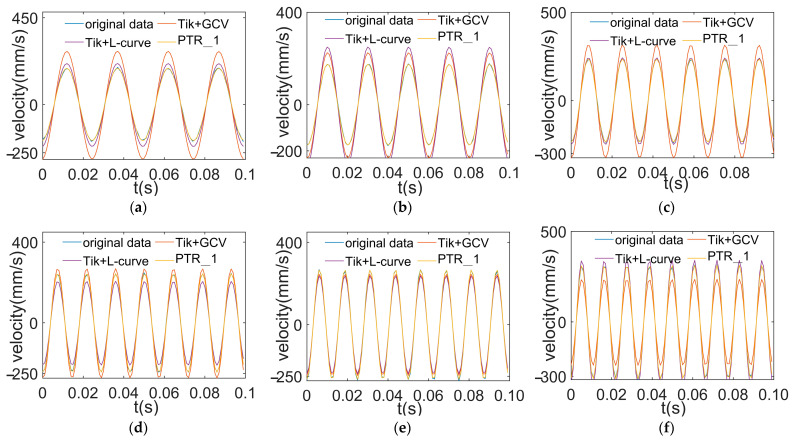
Reconstructed load at different frequencies using an initial value of 80 Hz. (**a**) Reconstructed excitation at 40 Hz; (**b**) reconstructed excitation at 50 Hz; (**c**) reconstructed excitation at 60 Hz; (**d**) reconstructed excitation at 70 Hz; (**e**) reconstructed excitation at 80 Hz; (**f**) reconstructed excitation at 90 Hz.

The reconstruction results using an initial value of 70 Hz are shown in Figure 14. This shows that the higher the image overlap, the better the reconstruction effect. The reconstruction results using an initial value of 80 Hz are shown in Figure 15. In the same vein, a higher degree of overlap is needed here as well.

From Figure 14 and Figure 15, it can be seen that all the regularization methods are able to approximately reconstruct the load signals at different frequencies. However, the reconstruction curves of the method presented in this paper are closer to the original data curves. This is especially apparent in the peak regions. This indicates that the method in this paper has higher accuracy in the load reconstruction process. Using the segmented Tikhonov regularization method enables us to eliminate noise and errors more effectively, thereby approximating the true load conditions more closely.

To evaluate the reconstruction accuracy of the method in this paper, the MRE and PRE are used to characterize it. The reasons for using the MRE as the primary parameter to validate the effectiveness of the method are as follows: Firstly, the MRE represents the average relative error between the reconstructed result and the actual value, providing an assessment of the overall accuracy of the reconstruction result. Compared to the error of individual samples, the MRE can provide a better representation of the fitting degree of the entire dataset. Secondly, during the evaluation of the reconstruction methods, it is typically desirable to understand the extent of the average deviation between the reconstructed result and the actual value. The MRE provides a unified way to quantify this deviation, enabling comparisons across different datasets and scenarios. By calculating the relative errors of all the samples and taking their average, we can obtain a global performance indicator, thereby gaining a better understanding of the performance of the reconstruction method. Thirdly, the MRE is less sensitive to outliers in the dataset, as it is based on relative error computation. This means that even if there are some outliers in the dataset, the MRE can still provide a robust assessment of the overall performance of the reconstruction method.

The excitations in the frequency band from 40 Hz to 90 Hz are reconstructed using the initial transfer function matrices and regularization parameters. The MRE is solved using Equation (18), as Table 5 shows. It can be seen that the smaller the values of the MRE, the better the overall reconstruction results, and vice versa. The PRE is solved using Equation (19), as Table 6 shows. It can be seen that the smaller the values of the PRE, the smaller the errors in the amplitude, and vice versa.

When the initial transfer function matrix of 70 Hz is used, the MRE and PRE of the PTR method are improved by 78.20% and 86.89%, respectively, compared with the traditional Tikhonov regularization method based on GCV. Similarly, the MRE and PRE of the PTR method are improved by 79.31% and 68.18%, respectively, compared with the traditional Tikhonov regularization method based on an L-curve. When the 80 Hz initial transfer function matrix is used, the MRE and PRE of the PTR method are improved by 80.21% and 86.12%, respectively, compared with the traditional Tikhonov regularization method based on GCV. Similarly, the MRE and PRE of the PTR method are improved by 88.00% and 76.02%, respectively, compared with the traditional Tikhonov regularization method based on an L-curve. The MRE and PRE of the PTR method are both improved compared with the traditional Tikhonov regularization method, indicating that the accuracy of the proposed method is superior to the traditional method.

To further illustrate the soundness of the PTR method, the reconstruction errors are analyzed for different numbers of segments. PTR_2 increments the number of segments based on the PTR_1 method by 1. Table 7 shows the MRE values. It can be seen that the smaller the MRE is, the more reasonable the choices of the number of segments are, and vice versa. Table 8 shows the PRE values. It can be seen that the smaller the PRE is, the more reasonable the choices of the number of segments are, and vice versa.

Comparing the PTR_2 method with the traditional Tikhonov regularization method, the MRE decreased by 62.73% and 68.15%, respectively. This indicates the PRT_2 method is still effective. Comparing the PTR_1 method with the traditional Tikhonov regularization method, the MRE increased by 70.97% and 60.92%, respectively. This indicates that over-segmentation can cause over-regularization. The instantaneous relative error peak may be caused by noise interference. The PTR_2 method simultaneously exhibits both the minimum and maximum values of the PRE, indicating that an increase in the number of segments can mean that the instantaneous relative error peak cannot be suppressed effectively. An increase in the number of segments can cause greater calculation complexity and inaccurate solutions for the regularization parameters. The results show that the accuracy and stability of the approximate solution can be balanced more reasonably. The performance of the PTR method is better than the traditional Tikhonov regularization method.

In summary, this method achieves low MREs and PREs at different frequencies, indicating its high accuracy in load reconstruction. Therefore, when the unknown response of a strain sensor is put into the load reconstruction model proposed in this paper, the small MRE of the load reconstruction result suggests the high precision of the strain sensor.

## 5. Conclusions

The research presented in this paper addresses the dynamic load reconstruction problem for standard beams using the proposed PTR method. The conclusions drawn from this study are multifaceted and hold significant implications for practical applications.

- According to the finite element simulation analysis, the load reconstruction problem based on cantilever beams is ill posed. This underscores the necessity for advanced numerical methods to address the complexity of the inverse problem.
- The experimental results on the cantilever beams demonstrate that the PTR method accurately reconstructs loads across different frequency signals. When the initial transfer function matrix at 70 Hz is known, the reconstructed MRE and PRE are 6.20% and 3.54%, respectively. When the initial transfer function matrix at 80 Hz is known, the reconstructed MRE and PRE are 5.86% and 3.73%, respectively. The condition numbers obtained for the modified transfer function matrices are all close to 1, indicating the reliability of the reconstruction results. Compared with the traditional Tikhonov regularization method, the PTR method exhibits significantly reduced MREs and PREs at different frequencies. Comparative analysis demonstrates that the PTR method is superior to the traditional Tikhonov regularization method.
- Future work will include studying the applicability of the PTR method to structures other than cantilever beams and exploring methods for load reconstruction using complex signals.

## Figures and Tables

**Table 1:** Condition numbers with different SNRs.

Freq./Hz	∞ dB	26 dB	20 dB
40	1.00	1.99 × 10^10^	1.66 × 10^11^
50	1.00	1.84 × 10^8^	7.55 × 10^8^
60	1.00	3.40 × 10^2^	3.67 × 10^6^
70	1.00	6.44 × 10^3^	2.98 × 10^5^
80	1.00	1.20 × 10^3^	8.80 × 10^3^
90	1.00	4.36 × 10^1^	1.93 × 10^7^

**Table 2:** MRE (%) and PRE (%) at different SNRs.

Freq./Hz	MRE				PRE			
	∞ dB	26 dB	20 dB	14 dB	∞ dB	26 dB	20 dB	14 dB
40	0.00	11.99	47.59	93.53	0.00	1.69	8.10	24.41
50	0.00	35.15	66.10	127.75	0.00	4.24	13.75	45.90
60	0.00	11.55	33.71	79.54	0.00	5.66	14.30	27.48
70	0.00	11.91	30.05	60.41	0.00	6.32	10.04	39.23
80	0.00	16.09	53.63	73.03	0.00	2.30	5.57	21.45
90	0.00	7.78	25.67	48.92	0.00	5.63	8.46	33.30
mean	0.00	15.74	42.79	80.53	0.00	4.31	10.04	31.96

**Table 3:** Waveform correlation.

Freq./Hz	Cor			
	∞ dB	26 dB	20 dB	14 dB
40	1.000	0.999	0.995	0.982
50	1.000	0.998	0.995	0.980
60	1.000	0.998	0.995	0.978
70	1.000	0.999	0.994	0.979
80	1.000	0.998	0.995	0.980
90	1.000	0.998	0.995	0.979
mean	1.000	0.998	0.995	0.979

**Table 4:** Segmentation parameters.

Freq./Hz	PTR_1			PTR_2		
	$\gamma_{1}$	K	$P_{k},Q_{k}$	$\gamma_{1},\gamma_{2}$	K	$P_{k},Q_{k}$
70	0.8	2	1–9 10–100	$\begin{array}{l} \gamma_{1}=0.8\\ \gamma_{2}=0.9 \end{array}$	3	1–9 10–14 15–100
80	0.8	2	1–99 100–100	$\begin{array}{l} \gamma_{1}=0.3\\ \gamma_{2}=0.8 \end{array}$	3	1–9 10–99 100–100

**Table 5:** MREs (%) of different regularization methods.

Freq./Hz		GCV	L-Curve	PTR
70	40	29.28	38.43	8.45
	50	56.08	22.63	5.73
	60	21.82	44.39	8.98
	70	9.05	9.22	5.71
	80	19.98	12.69	3.40
	90	34.40	52.55	4.95
mean		28.44	29.98	6.20
80	40	48.02	14.93	4.58
	50	29.99	43.34	4.24
	60	55.96	16.55	7.23
	70	16.51	16.06	6.47
	80	8.10	9.97	4.27
	90	27.28	9.68	6.01
mean		29.61	18.42	5.86

**Table 6:** PREs (%) of different regularization methods.

Freq./Hz		GCV	L-Curve	PTR
70	40	25.54	37.66	3.03
	50	59.46	21.30	2.67
	60	15.42	43.28	3.15
	70	7.63	8.84	2.31
	80	16.58	13.15	5.39
	90	37.40	52.80	4.68
mean		27.01	29.50	3.54
80	40	44.88	13.30	4.52
	50	28.51	42.48	3.54
	60	33.30	3.18	4.12
	70	16.10	15.58	5.05
	80	10.27	10.35	3.05
	90	27.39	8.52	3.43
mean		26.88	15.56	3.73

**Table 7:** MREs (%) for different numbers of segments.

Freq./Hz		GCV	PTR_1	PTR_2
70	40	29.28	8.45	4.46
	50	56.08	5.73	25.58
	60	21.82	8.98	17.79
	70	9.05	5.71	5.93
	80	19.98	3.40	5.69
	90	34.40	4.95	4.12
mean		28.44	6.20	10.60
80	40	48.02	4.58	4.93
	50	29.99	4.24	7.07
	60	55.96	7.23	20.83
	70	16.51	6.47	8.18
	80	8.10	4.27	4.00
	90	27.28	6.01	3.44
mean		29.61	5.86	9.43

**Table 8:** PREs (%) for different numbers of segments.

Freq./Hz		GCV	PTR_1	PTR_2
70	40	25.54	3.03	4.15
	50	59.46	2.67	12.37
	60	15.42	3.15	2.35
	70	7.63	2.31	3.42
	80	16.58	5.39	2.55
	90	37.40	4.68	2.57
mean		27.01	3.54	4.57
80	40	44.88	4.52	3.39
	50	28.51	3.54	2.74
	60	33.30	4.12	8.39
	70	16.10	5.05	3.68
	80	10.27	3.05	6.78
	90	27.39	3.43	3.76
mean		26.88	3.73	4.67

## Data Availability

The data used to support the findings of this study are available from the first author upon request.

## References

[1] Nyssen F., Tableau N., Lavazec D., Batailly A. (2020). Experimental and Numerical Characterization of a Ceramic Matrix Composite Shroud Segment under Impact Loading. J. Sound Vib..

[2] Wang Q., Xu F., Guo W., Gao M. (2022). New Technique for Impact Calibration of Wide-Range Triaxial Force Transducer Using Hopkinson Bar. Sensors.

[3] Du L., Jiang W., Luo Z., Song H., Yang L., Li H. (2022). Multi FBG Sensor-Based Impact Localization with a Hybrid Correlation Interpolation Method. Meas. Sci. Technol..

[4] Li Q., Hou M., Cao H. (2023). Online Identification of Milling Loads Using Acceleration Signals. Int. J. Adv. Manuf. Technol..

[5] Sanchez J., Benaroya H. (2014). Review of Force Reconstruction Techniques. J. Sound Vib..

[6] Liu R., Dobriban E., Hou Z., Qian K. (2022). Dynamic Load Identification for Mechanical Systems: A Review. Arch. Comput. Methods Eng..

[7] Liu C.S., Kuo C.L., Chang C.W. (2021). Recovering External Loads on Vibrating Euler–Bernoulli Beams Using Boundary Shape Function Methods. Mech. Syst. Signal Process..

[8] Zhao M., Wu G., Wang K. (2022). Comparative Analysis of Dynamic Response of Damaged Wharf Frame Structure under the Combined Action of Ship Collision Load and Other Static Loads. Buildings.

[9] Zhang E., Antoni J., Feissel P. (2012). Bayesian Force Reconstruction with an Uncertain Model. J. Sound Vib..

[10] Yan G., Sun H. (2019). A Non-Negative Bayesian Learning Method for Impact Load Reconstruction. J. Sound Vib..

[11] Li Q., Lu Q. (2018). Time Domain Force Identification Based on Adaptive ℓq Regularization. J. Vib. Control.

[12] Prawin J., Rao A.R.M. (2018). An Online Input Load Time History Reconstruction Algorithm Using Dynamic Principal Component Analysis. Mech. Syst. Signal Process..

[13] Jiang W.S., Wang Z.Y., Lv J. (2018). A Fractional-Order Accumulative Regularization Filter for Force Reconstruction. Mech. Syst. Signal Process..

[14] Pallekonda R.B., Nanda S.R., Dwivedy S.K., Kulkarni V., Menezes V. (2018). Soft Computing Based Force Recovery Technique for Hypersonic Shock Tunnel Tests. Int. J. Struct. Stab. Dyn..

[15] Cumbo R., Mazzanti L., Tamarozzi T., Jiranek P., Desmet W., Naets F. (2021). Advanced Optimal Sensor Placement for Kalman-Based Multiple-input Estimation. Mech. Syst. Signal Process..

[16] Lourens E., Fallais D. (2019). Full-Field Response Monitoring in Structural Systems Driven by a Set of Identified Equivalent Loads. Mech. Syst. Signal Process..

[17] Liu L., Zhang Y., Lei Y., Yang N. (2023). Identification of Distributed Dynamic Loads in Gradually Varying Two Spatial Dimensions Based on Discrete Cosine Transform and Kalman Filter with Unknown Inputs. J. Aerosp. Eng..

[18] Zou D., Zhao H., Liu G., Ta N., Rao Z. (2019). Application of Augmented Kalman Filter to Identify Unbalance Load of Rotor-Bearing System: Theory and experiment. J. Sound Vib..

[19] Tian Y., Zhang Y. (2022). A Comprehensive Survey on Regularization Strategies in Machine Learning. Inf. Fusion.

[20] Lu C., Zhu L., Liu J., Meng X., Li K. (2023). The Least Squares Time Element Method Based on Wavelet Approximation for Structural Dynamic Load Identification. Int. J. Comput. Methods.

[21] Miao B., Zhou F., Jiang C., Luo Y., Chen H. (2020). A Load Identification Application Technology Based on Regularization Method and Finite Element Modified Model. Shock Vib..

[22] Tang Z., Zhang Z., Xu Z., He Y., Jin J. (2022). Load Identification with Regularized Total Least-Squares Method. J. Vib. Control.

[23] Sun X., Cui J., Chen Y., Tan J. (2022). A Novel Method for Identifying Rotor Unbalance Parameters in the Time Domain. Meas. Sci. Technol..

[24] He Z., Lin X., Li E. (2018). A Novel Method for Load Bounds Identification for Uncertain Structures in Frequency Domain. Int. J. Comput. Methods.

[25] Miao B., Zhou F., Jiang C., Chen X., Yang S. (2018). A Comparative Study of Regularization Method in Structure Load Identification. Shock Vib..

[26] Wang L.J., Gao X., Xie Y.X., Fu J.J., Du Y.X. (2021). A New Conjugate Gradient Method and Application to Dynamic Load Identification Problems. Int. J. Acoust. Vib..

[27] Aucejo M., De Smet O. (2018). An Iterated Multiplicative Regularization for Load Reconstruction Problems. J. Sound Vib..

[28] Zheng H., Zhang W. (2019). A Mixed Regularization Method for Ill-Posed Problems. Numer. Math. Theory Methods Appl..

[29] Chang X., Yan Y., Wu Y. (2019). Study on Solving the Ill-Posed Problem of Load Reconstruction. J. Sound Vib..

[30] Chen Z., Fang Y., Kong X., Deng L. (2023). Identification of Multi-Axle Vehicle Loads on Beam Type Bridge Based on Minimal Residual Norm Steepest Descent Method. J. Sound Vib..

[31] Yang J., Hou P., Yang C., Zhou Y., Zhang G. (2023). Investigation on the Moving Load Identification for Bridges Based on Long-Gauge Strain Sensing and Skew-Laplace Fitting. Smart Mater. Struct..

[32] Pan C., Yu L. (2019). Identification of External Forces via Truncated Response Sparse Decomposition under Unknown Initial Conditions. Adv. Struct. Eng..

[33] Zhang Z., Huang W., Liao Y., Song Z., Shi J., Jiang X., Shen C., Zhu Z. (2022). Bearing Fault Diagnosis via Generalized Logarithm Sparse Regularization. Mech. Syst. Signal Process..

[34] Qiao B., Ao C., Mao Z., Chen X. (2020). Non-Convex Sparse Regularization for Impact Load Identification. J. Sound Vib..

[35] Liu J., Qiao B., Wang Y., He W., Chen X. (2023). Non-Convex Sparse Regularization via Convex Optimization for Impact Load Identification. Mech. Syst. Signal Process..

[36] Liu J., Qiao B., He W., Yang Z., Chen X. (2020). Impact Load Identification via Sparse Regularization with Generalized Minimax-Concave Penalty. J. Sound Vib..

[37] Tran H., Inoue H. (2018). Development of Wavelet Deconvolution Technique for Impact Load Reconstruction: Application to Reconstruction of Impact Load Acting on a Load-Cell. Int. J. Impact Eng..

